# Trends in contraceptive use, unmet need and associated factors of modern contraceptive use among urban adolescents and young women in Guinea

**DOI:** 10.1186/s12889-020-09957-y

**Published:** 2020-12-01

**Authors:** Sidikiba Sidibé, Alexandre Delamou, Bienvenu Salim Camara, Nafissatou Dioubaté, Hawa Manet, Alison M. El Ayadi, Lenka Benova, Seni Kouanda

**Affiliations:** 1 Institut Africain de Santé Publique (IASP/USTA) of the University Saint Thomas D’Aquin, Ouagadougou, Burkina Faso; 2 CEA-PCMT_Faculty of Sciences and Health Techniques, Gamal Abdel Nasser University of Conakry, Conakry, Guinea; 3 National Training and Research Centre in Rural Health of Maferinyah, Forecariah, Guinea; 4 Department of Obstetrics, Gynecology and Reproductive Sciences, Bixby Center for Global Reproductive Health, University of California San Francisco, San Francisco, CA USA; 5 Sexual and Reproductive Health Group, Department of Public Health, Institute of Tropical Medicine, Antwerp, Belgium; 6 Institut Africain de Santé Publique (IASP), Ouagadougou, Burkina Faso

**Keywords:** Contraception, Family planning, Unmet need, Adolescents and young adults, Guinea DHS, Health disparities

## Abstract

**Background:**

In Guinea, high fertility among adolescents and young women in urban areas remains a public health concern. This study describes trends in contraceptive use, unmet need, and factors associated with the use of modern family planning (FP) methods among urban adolescents and young women in Guinea.

**Methods:**

We used four Guinea Demographic and Health Surveys (DHS) conducted in 1999, 2005, 2012, and 2018. Among urban adolescents and young women (15–24 years), we examined trends over time in three key indicators: 1. Modern Contraceptive use, 2. Unmet need for FP and 3. Modern contraceptive use among those in need of FP (demand satisfied). We used multivariable logistic regression to examine association between socio-demographic factors and modern FP use on the most recent DHS dataset (2018).

**Results:**

We found statistically significant changes over the time period examined with an increase in modern contraceptive use (8.4% in 1999, 12.8% in 2018, *p* < 0.01) and demand satisfied (29.0% in 1999, 54.1% in 2018, *p* < 0.001), and a decrease in unmet need for FP (15.8% in 1999, 8.6% in 2018, *p* < 0.001). Factors significantly associated with modern FP use were; young women aged 20–24 years (AOR 2.8, 95% CI: 1.9–4.1), living in urban areas of Faranah (AOR: 2.6, 95% CI: 1.1–6.5) and Kankan (AOR: 3.6, 95% CI: 1.7–7.8), living in households in the middle (AOR: 7.7, 95% CI: 1.4–42.2) and richer wealth quintiles (AOR: 6.3, 95% CI: 1.0–38.1). Ever-married women (AOR: 0.5, 95% CI: 0.3–0.9) were less likely to use modern FP methods than never married as were those from the Peulh (0.3, 95% CI: 0.2–0.4) and Malinke (0.5, 95% CI: 0.3–0.8) ethnic groups compared to Soussou ethnic group.

**Conclusion:**

Despite some progress, efforts are still needed to improve FP method use among urban adolescent and young women. Age, administrative region, wealth index, marital status, and ethnic group are significantly associated with modern FP use. Future policies and interventions should place emphasis on improving adolescents’ reproductive health knowledge, increasing FP availability and strengthening provision. Efforts should target adolescents aged 15–19 years in particular, and address disparities between administrative regions and ethnic groups, and health-related inequalities.

## Background

Adolescent fertility remains high, particularly in Africa, despite some improvements over the past two decades. In 2018, the birth rate among adolescent girls (15–19 years) worldwide was 44 births per 1000 women, a 22% decrease with respect to 2000 (56 births per 1000 women). In the same year, the highest regional adolescent birth rate (101 births per 1000 females) was observed in sub-Saharan Africa [1]. Adolescent childbearing remains a major public health concern, mostly in low- and middle-income countries [2–4] due to high levels of unwanted fertility (including shortly-spaced births) among young women [5]. Approximately 21 million girls aged 15–19 years in developing countries become pregnant every year and 12 million of them give birth [6]. It is estimated that 90% of the over six million annual unplanned pregnancies, either unwanted or mistimed, among adolescent girls in sub-Saharan Africa, Latin America and the Caribbean, and South Central and Southeast Asia are due to non-use of a modern method of contraception [6] .

In sub-Saharan Africa, unwanted adolescents pregnancies [7–9] have adverse consequences for the women concerned, with poverty, education dropout and subsequent lower educational attainment holding back their personal development. But women should also have rights to reproductive health beyond their potential contribution to economic growth [9–13].

In Guinea, almost half of the population is under 24 years old [14] and the age-specific fertility rate remains high among adolescent girls (15–19 years) (146 births per 1000 women). The fertility rate among young women aged 20–24 years (202 births per 1000 women) is higher than the overall rate for sub-Saharan Africa [15, 16]. In 2012, 67% of first births in Guinea were to mothers below the age 20 years, and this percentage has remained stable since 1999 [5] . While the percentage of sexually active adolescent girls and young women (15–24 years) using any contraceptive increased from 7.2% in 1999 to 11.4% in 2018, the level remains very low [17, 18]. This is evidenced by the fact that 20% of females in this age group had an unmet need for family planning in 2018 [17]. In addition, the prevalence of modern FP methods among all women of reproductive age remains relatively low 15.4% [17] and this is even the case for women in urban areas with better access to FP services and higher socio-economic status. According to the National Institute of Statistics, 36% of the total Guinean population lives in urban areas, but the proportion of adolescents and youths is higher in urban areas (24.8%) than in rural areas (16.1%) [19]. Modern contraceptive prevalence among urban adolescent and young women aged 15–24 years remain low (11.07%) despite the fact that more than 84% of health personnel (doctors, nurses and midwives) work in urban areas [20]. This represents an opportunity to increase the availability and use of modern contraceptives among the young urban population.

Prior studies have identified the determinants of contraceptive use among women of childbearing age (15–49 years) [21–24] and the factors associated with this use in young sexually active women through a secondary analysis of Demographic and Health Surveys (DHS) from selected countries in Sub-Saharan Africa [7, 25–29]. However, the trends over time and the factors influencing contraceptive use among adolescents and young women in Guinean urban areas are insufficiently understoodSuch is information critically important for developing strategies and interventions to increase contraceptive use. More generally, better knowledge better knowledge would help to address the ongoing challenges threatening the sexual and reproductive health of adolescents and young people in urban areas, including human immunodeficiency virus (HIV) and sexually transmitted infections [30, 31].

The Ministry of Health of Guinea recognizes the existence of specific challenges in meeting the contraceptive needs of Guinean female adolescents and young women However, young people’s concerns were not prioritized in FP policy development until recently and the existing FP services are often unsuited to their needs [32]. Hence, in 2015, the Government of Guinea drew up the 2015–2019 Strategic Plan for the Health and Development of Adolescents and Youth, and in September 2018 established a budgeted 2019–2023 action plan for FP [32, 33]. One of outlines of this plan was advocacy with decision-makers for free FP services, in particular for adolescents and young people from 2019 to 2023 [32]. In Guinea, health sector funding has focused on increasing service provision, however, concurrent operations and action research initiatives are also needed to guide efforts to stimulate demand and improve service provision [15].

The objective of this paper is to analyze the trends in contraceptive use, unmet need, and the factors associated with modern contraceptive use among urban adolescents and young women in Guinea, using data from four Demographic and Health Surveys (DHS) conducted between 1999 to 2018.

## Methods

### Data and population

Data for this study were extracted from four Guinea Demographic and Health Surveys (DHS) conducted in 1999, 2005, 2012, and 2018. The DHS are nationally representative household surveys that collect data on a wide range topics relating to reproductive, maternal and child health such as fertility, health-seeking behavior, and FP use. A two-stage stratified cluster design was employed in survey sampling based on a list of enumeration areas (EAs) from the 1999–2018 General Population Census of the Republic of Guinea. All women age 15–49 years who were permanent residents or visitors in sampled households the night before the survey were eligible for the women’s DHS survey. In this study sample, we included sexually active urban dwelling adolescent and young women aged 15–24 years at the time of the surveys.

The surveys thus covered the populations living in urban survey strata of all eight administrative regions of Guinea (Conakry, Boke urban, Faranah urban, Kankan urban, Kindia urban, Labe urban, Mamou urban, and N’Zérékoré urban strata). Women with missing data on outcome variables were excluded from the analyses.

All reproductive health variables are based on women’s self-reports. During analyses, we further disaggregated into respondent age groups 15–19 years and 20–24 years.

### Outcomes variables

The main outcome variable was current modern FP use, and was coded as a binary variable: ‘yes’ for respondents who reported using a modern contraceptive method at the time of survey, and ‘no’ for those not using any modern method, including those using traditional contraceptive methods. Modern FP methods were defined as: intrauterine device (IUD), implants, injectable, the pill, condoms (male and female), and sterilization (male and female). Traditional FP methods were defined as: lactational amenorrhea (LAM), periodic abstinence, withdrawal, and folkloric methods (gris-gris).

The secondary outcomes included current unmet need for FP and FP demand satisfied.

Unmet need for FP is defined as women wishing to limit or space pregnancies but not using any FP method among all sexually active urban adolescent and young women. This includes respondents who are married or unmarried but sexually active considered fecund but neither pregnant nor in postpartum amenorrhea and who wants to space their next birth by at least 2 years or limit their pregnancies but are not using a modern method of contraception; and those current pregnancy or last delivery was unwanted for at least 2 years [34]. This outcome was generated from a constructed Guinea 2018 Demographic Health Survey variable.

Satisfied demand for contraception was defined as women using any modern FP method among women in need of FP.

### Independent variables

The independent variables included socio-demographic characteristics (age group at time of survey, region of residence, marital status at time of survey, ethnicity, religion, educational level, and household wealth quintile). Due to the very small sample size of poorest and poorer wealth quintile, we categorized household wealth index into four groups, merging the poorest two quintiles. Educational attainment included no education, primary, secondary and higher levels. Religion had two categories, Muslim and Christian/other. Marital status either never-married or ever-married. Ethnicity included four groups (Soussou, Peulth, Maninké, and Other). Region was categorized as Conakry, Kindia, Boké, Mamou, Labé, Kankan, Faranah, and Nzerekore..

### Measures

We categorized respondents based on the need for FP and the use of modern FP methods as classified by the DHS [34]: 1) women not exposed to pregnancy, i.e. unmarried and no sexual intercourse in the 30 days preceding the survey; 2) Women exposed to pregnancy, but with no unmet need for FP (individuals who want to become pregnant within the next 2 years); 3) Women exposed to pregnancy and using FP; and 4) Women exposed to pregnancy, who do not want to be pregnant but are not using FP (unmet need for FP). (Table 1).

**Table 1:** Key indicators of family planning used in analysis

Indicator	Numerator	Denominator
% Using of a modern FP method among all	Women using a modern FP method	All sexually active urban girls and young women
% Unmet need for FP among all women	Women in need of FP but not using any FP method	Women in need of FP
% using a modern FP method among women in need of FP (demand satisfied)	Women using a modern FP method	Women in need of FP (Groups 3 + 4)

### Statistical analysis

We described the sample using socio-demographic characteristics (age groups 15–19 years and 20–24 years, region of residence, marital status at time of survey, ethnicity, religion, educational level, and household wealth quintile category: poorest/poorer, middle, richer, richest).

We estimated the three indicators in Table 1 and their 95% confidence intervals for all four surveys. We visually plotted the trends over time for three key indicators and used Pearson’s Chi-squared to test the differences between estimates on subsequent surveys, e.g., 1999 and 2005. Pearson’s Chi-squared test was used to assess the difference across surveys in the levels of the three indicators. This comparison of proportions was applied with values of *p* < 0.05 taken as significant.

The determinants of modern FP methods use among adolescent and young women in urban Guinea were analyzed through a logistic regression using the most recent DHS dataset (2018). Multicollinearity was checked first and then socio-demographic then variables were included in the model as covariates. The multivariate logistic regression was fitted to predict associated factors of modern contraceptive use in the presence of selected covariates. Independent variables included in the model were derived from the literature review [23, 28, 35, 36] and the 2018 DHS questionnaire as determinants of contraceptive use. All the analyses incorporated an adjustment for sampling design using sampling weights, clustering and stratification. Adjusted odds ratios (AOR) were then calculated with 95% confidence intervals. The data were analyzed using Stata 16.0 software (StataCorp, College Station, Tx USA).

## Results

### Characteristics of the sample

We included 1026 urban women aged 15–24 in 1999, 1034 aged 15–24 in 2005, 1650 aged 15–24 in 2012 and 1876 aged 15–24 in 2018. Table 2 shows the profile of the urban adolescent girls and young women in our analysis sample for each DHS. The percentage of respondents with a secondary or higher level of education more than doubled from 23.9% in 1999 to 52.9% in 2018. The proportion never married urban adolescent and young women increased from 53.8% in 1999 to 67.4% in 2018. In terms of the household wealth index, more than 90% were living in households from richer or richest wealth quintiles. However, over time, the proportion in poorest and poor quintile increased seven fold while the proportion in the middle quintile almost doubled. There were no significant differences over time in the region, religion, and ethnicity profiles of the urban samples between 1999 and 2018 (Table 2).

**Table 2:** Descriptive characteristics of urban adolescents and young women (15–24 years old), Guinea by survey year

Socio-demographic characteristics	Variables	1999 DHS		2005 DHS		2012 DHS		2018 DHS	
		***n*** = 1026 Weighted ***n*** = 950		***n*** = 1034 Weighted ***n*** = 1095		***n*** = 1650 Weighted: ***n*** = 1565		***n*** = 1876 Weighted ***n*** = 1916	
		%	95% CI	%	95% CI	%	95% CI	%	95% CI
Age group (years)	15–19	57.5	53.8–61.2	59.4	56.2–62.5	54.3	51.6–56.9	58.8	56.2–61.4
	20–24	42.5	38.8–46.2	40.7	37.5–43.9	45.7	43.1–48.4	41.2	38.6–43.8
Administrative region	Boke urban	NA	NA	9.1	7.3–11.4	9.9	8.0–12.3	7.3	5.9–9.1
	Conakry	NA	NA	47.9	42.5–53.4	58.9	53.3–64.3	46.2	41.5–51.0
	Faranah urban	NA	NA	5.3	4.2–7.3	5.7	3.8–8.5	5.9	4.4–7.7
	Kankan urban	NA	NA	5.7	4.3–7.4	5.2	4.1–6.7	8.0	6.6–9.7
	Kindia urban	NA	NA	8.8	6.1–12.5	9.1	6.2–13.0	13.8	11.1–17.1
	Labe urban	NA	NA	3.5	2.6–4.7	1.6	1.0–2.5	2.9	1.9–4.4
	Mamou urban	NA	NA	2.4	1.7–3.5	1.9	1.3–2.7	3.5	1.7–7.1
	N’Zérékoré urban	NA	NA	17.1	11.6–24.3	7.7	6.0–9.9	12.4	8.8–17.4
Education level	No Education	46.9	43.5–50.7	39.7	35.9–43.7	26.2	22.8–30.0	28.9	26.1–31.1
	Primary	29.2	26.2–32.3	21.8	19.1–24.8	20.9	18.2–23.9	18.2	15.3–20.7
	Secondary	22.3	19.2–25.8	37.8	33.4–42.4	44.7	41.1–48.3	46.0	41.9–48.4
	Higher	1.6	1.0–2.7	0.6	0.3–1.3	8.2	6.2–10.7	6.9	7.0–10.4
Religion	Muslim	90.5	87.2–92.9	87.4	81.2–91.7	91.7	89.1–93.7	88.8	81.2–91.9
	Christian and other	9.5	7.1–12.8	12.7	8.3–18.8	8.3	6.3–10.9	11.2	8.1–18.8
Marital status	Never married	53.8	49.9–57.7	59.0	54.3–63.6	64.9	60.6–69.0	67.4	65.1–71.0
	Ever married	46.2	42.3–50.2	41.0	36.4–45.7	35.1	31.1–39.4	32.6	28.9–34.9
Ethnicity	Soussou	29.5	24.4–35.0	25.6	19.9–31.4	28.6	22.7–35.3	29.5	24.7–34.8
	Peulh	28.1	23.4–33.5	33.7	27.7–40.2	31.2	24.9–38.2	25.7	21.5–30.3
	Malinké	30.4	26.4–34.8	26.2	22.4–30.3	29.0	25.6–32.8	30.6	26.6–35.0
	Other	12.0	9.3–14.9	14.9	10.7–20.4	11;3	8.8–14.3	14.2	9.7–20.4
Household wealth index (quintiles)	Poorest	0.2	0.0–1.4	0.6	0.2–2.1	0.5	0.2–1.3	0.4	0.2–2.2
	Poorer	0.2	0.0–0.8	1.2	0.5–2.7	0.8	0.4–2.0	2.4	1.1–6.7
	Middle	3.7	2.6–5.5	4.7	2.9–7.3	2.7	1.8–4.1	6.1	4.0–8.0
	Richer	28.1	23.6–33.0	27.5	22.8–32.6	33.3	28.5–38.4	35.9	28.6–37.5
	Richest	67.8	62.9–72.4	66.1	60.6–71.2	62.7	57.3–67.8	55.2	52.6–63.2

### Trends in FP method use among adolescents and young women in urban areas

**Fig. 1:**
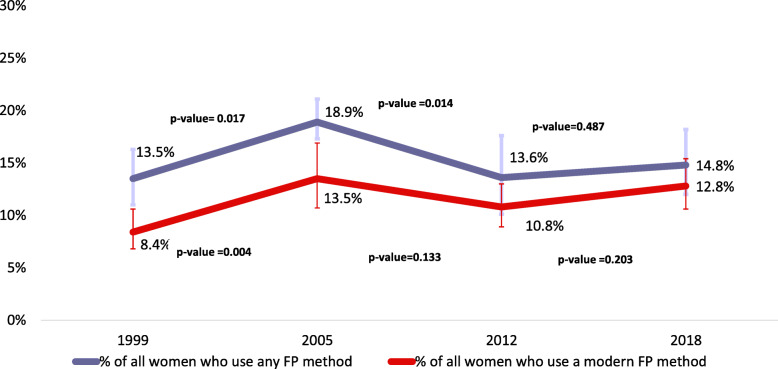
Trends in use of FP methods among adolescents and young women in urban areas Guinea

Figure 1 showed that the largest and statistically significant increase in FP method use was observed between the first two surveys (1999–2005) with a 5.4% increase for any FP method use (*p* = 0.017) and a 5.1% increase for modern FP method use (*p* = 0.004). In comparison, for the second phase (2005–2012) there was a significant 5.3% decline in any FP method use (*p* = 0.014). However, the slight 2.3% decrease in modern FP method use among the urban adolescent was not statically significant (*p* > 0.05). During the third phase (2012–2018), the percentage of both indicators increased slightly, but these increases were not statistically significant (*p* > 0.05) (Fig. 1).

### Time-trends in unmet need and modern FP method use among urban adolescents and young women in need of FP in Guinea, by DHS

Unmet need for FP among urban adolescents and young women decreased by 7.2 percentage points over the period (*p* < 0.001) from 15.8% in 1999 to 8.6% in 2018. There was a 7.2 percentage point decrease of unmet need during the period (*p* < 0.001).

**Fig. 2:**
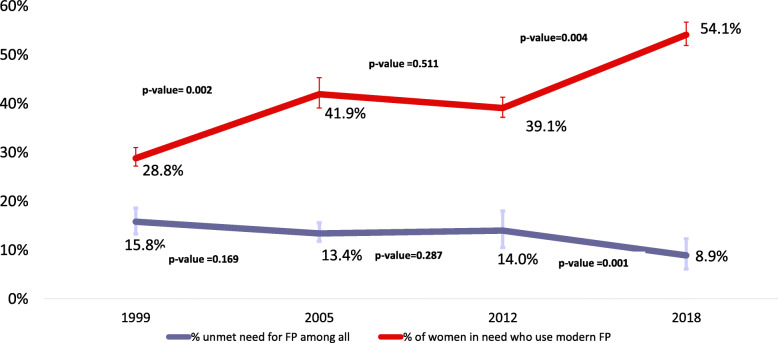
Time-trends in unmet need and demand satisfied among urban adolescents and young women in need of FP in Guinea, by DHS

Across the four DHS surveys, among the study participants in need of contraceptives, the percentage using modern FP methods increased from 29.0% (95% CI: 23.9–34.7) in 1999 to 54.1% (95% CI: 48.3–59.748.3–59.7) in 2018. The proportion of urban adolescents and young women in need of contraceptives who used modern FP showed the largest and statistically significant increase during the first phase (1999–2005) with a 13.1% increase (*p* < 0.001) and during the third phase (2012–2018), with a 15.0% increase (*p* < 0.001). In comparison, during the second phase, the proportion fell by 2.8% from 41.9% in 2005 to 39.1% in 2012, but the decrease but is not statistically significant (*p* = 0.51) (Fig. 2).

### Factors associated with use of modern FP methods (DHS 2018)

In the bivariate analysis, we found that among young all women aged 15–24 years, the odds of modern FP use were significantly higher among those from Boke and Kankan, those with higher education, those from the Peulh ethnic group, and those with middle and richer quintiles, compared to the respective reference groups. In analyses incorporating possible confounding variables, only age group, location, and household wealth index were associated with significant differences in modern FP method use. Compared with women aged 15–19 years, the odds of using modern FP methods were 2.8 times (AOR 2.8; 95% CI: 1.9–4.1) higher among women aged 20–24 years. Adolescents and young women living in urban areas of Faranah and Kankan had 2.6 times (AOR: 2.6; 95% CI: 1.1–6.5) and 3.6 times (AOR: 3.6; 95% CI: 1.7–7.8) higher odds of modern FP method use than women living in Conakry, respectively. The odds of using modern FP methods were 7.7 times (AOR: 7.7; 95% CI: 1.4–42.2) and 6.3 times (AOR: 6.3; 95% CI: 1.0–38.1) higher among adolescents from household in the middle and richer wealth quintiles compared to those from the poorer quintiles, respectively. Ever-married were 50% (AOR: 0.5; 95% CI: 0.3–0.9) less likely to use modern FP methods than never-married women. Women from the Peulh and Malinke ethnic groups were 70% (0.3; 95% CI: 0.2–0.4) and 50% (0.5; 95% CI: 0.3–0.8) less likely respectively, to use modern FP method than women from the Soussou ethnic group (Table 3).

**Table 3:** Current use of modern FP methods with regard to socio-demographic characteristics among urban adolescents and young women. Bivariate analysis and Logistic regression model with adjusted odds ratios, Guinea Demographic and Health Survey 2018 (*n* = 1876)

Socio-demographic characteristics	Variable	Bivariate				Multivariate Analysis			
		OR	95% CI		***p***-value	AOR	95% CI		Walid test
			Lower	Upper			Lower	Upper	***p***-value
**Age group (years)**	15–19	1				1			
	20–24	2.4	1.7	3.5	< 0.001	2.8	1.9	4.1	< 0.001*
**Administrative region**	Conakry	1				1			
	Boke urban	0.5	0.3	0.9	0.039	0.6	0.3	1.2	0.125
	Faranah urban	1.4	0.7	2.9	0.318	2.6	1.1	6.5	0.04*
	Kankan urban	2.1	1.1	3.9	0.018	3.6	1.7	7.8	0.001*
	Kindia urban	0.8	0.4	1.7	0.569	0.9	0.4	2	0.851
	Labe urban	0.3	0.1	1.4	0.131	0.7	0.1	4.1	0.718
	Mamou urban	0.6	0.3	1.2	0.184	1.6	0.8	3.2	0.231
	N’Zérékoré urban	1.0	0.6	1.8	0.948	1.5	0.7	3.5	0.312
**Education level**	No education	1				1			
	Primary	0.8	0.6	1.3	0.489	0.9	0.5	1.4	0.529
	Secondary	0,9	0.7	1.4	0.961	1.0	0.7	1.5	0.993
	Higher	2.4	1.5	4.1	0.001	1.8	0.9	3.2	0.063
**Religion**	Muslim	1				1			
	Christian and other	1.5	0.9	2.3	0.068	1.0	0.4	2.1	0.923
**Marital status**	Never married	1				1			
	Ever married	0.7	0.5	1.1	0.115	0.5	0.3	0.9	0.021*
**Ethnicity**	Sousou	1				1			
	Peulh	0.3	0.2	0.5	< 0.001	0.3	0.2	0.4	< 0.001*
	Malinké	0.7	0.4	1.2	0.208	0.5	0.3	0.8	0.006*
	Other	1.1	0.6	1.7	0.854	0.7	3.3	1.7	0.462
**Household Wealth index (quintiles)**	Poorer/poorest	1				1			
	Middle	8.5	1.5	27.0	0.016	7.7	1.4	42.2	0.019*
	Richer	5.6	1.1	17.9	0.041	6.3	1.1	38.1	0.045*
	Richest	5.0	1.0	15.6	0.047	5.2	0.9	31.8	0.073

*OR* Crude odd ratio, *AOR* Adjusted odds ratio. *p-value = Evidence statistically significant.

## Discussion

To our knowledge, this study is one of the first to assess FP indicators in adolescents and young women in Guinea. It shows that despite a significant increase in the demand for and use of FP methods from 1999 to 2018, FP use among urban adolescents and young women remains low in Guinea. Predictors of FP use among these groups include being in the older age group [20–24], in the middle and higher wealth quintiles and living in the region of Kankan or Faranah. However, being married or belonging to the Malinke or Peulh ethnic group is associated with lower use of contraceptive methods.

Our findings from this secondary analysis of trends and factors associated with the use of modern contraceptive methods among adolescents and young women aged 15–24 years in Guinea have important policy and practice implications for the national contraceptive program focusing on adolescents and young women aged 15–24 years, and adolescent sexual and reproductive health strategies. Four out of five health personnel work in urban areas to serve just 38% of the population and FP services are integrated into all public health facilities [20]. Despite this easy access to health care in urban areas, the prevalence of modern FP method use among urban adolescent and young women aged 15–19 years (8.6%) is relatively low compared to the 14.6% reported in Ghana in 2015 [35].

### Trends in contraceptive use among adolescents and young women in urban areas

The trends in the use of modern FP methods among adolescent girls and young women show significant variation by period, with an increase from 1999 to 2018.

At the same time, an unmet need for FP decreased among all study participants over the time period examined. Hence, the proportion of demand satisfied among adolescents and young women also increased significantly over time from 1999 to 2018. These findings could help health authorities and their partners to support interventions aiming at improving young women’s reproductive health in Guinea. They point up the progress in the use of modern FP methods and challenges to be addressed. These specific challenges to meeting the contraceptive needs among the Guinean adolescents and young women are also recognized by the MoH [15]. Other studies have also reported failures to meet the contraceptive needs of adolescents and to achieve family planning goals of adolescents [36–38]. Satisfied demand for modern FP methods remains at 51.7% in the urban areas overall and at 47.0% for adolescents and young women aged 15–19 years and 46.7% for those aged 20–24 years [17].

### Factors associated with the use of modern FP methods

This study demonstrates that age group is significantly associated with the use of modern FP methods. Indeed, according to our findings, the odds ratios for use of a modern FP method are higher among young women (aged 20–24 years) than among adolescents (aged 15–19 years). This may be due to the fact that beyond 18 years, women become independent and are more likely to make decisions about choosing a modern contraceptive method or not. In addition, they are more sexually active, more educated and have more access to information on contraceptive methods. Similar findings were reported by Nyarko, S. H. in 2015 [35] and Patton G.C. et Al in 2016 [39]. The context of rigid social norms is another factor, along with the greater decision-making capacity and maturity of older young women to engage in decision making about their own health care including contraception, since they know more about the different types of FP method and their advantages, than to younger adolescents who may be less well-informed [23, 35, 39].

This study showed that urban adolescents and young women in the middle and richer wealth categories are more likely to use modern FP methods than those in the poorer quintiles. Studies conducted in different African countries [24, 28, 29, 40] have obtained similar results.

However, in this study, ever-married young women are less likely to use modern FP methods than those who are never-married. Others studies conducted in Ghana, Tanzania and Zimbabwe have also a similar significant relationship between modern FP methods use and adolescent marital status [35, 41]. Moreover, among those married or living with a partner at the time of the surveys and who were current users of FP methods, more than 82% of adolescents and 71% of young women made their own decision about FP [17]. Contrary to our findings, other studies have found a significant relationship between being married and using a modern FP method with adolescents and young women who are married or living with a partner being more likely to use modern FP methods than those who are not married (or do not have a partner) [35, 41]. Contrasting findings were reported by Nketiah-Amponsah et al. and Okech et al. [28, 42] who found no significant relationship between the two.

In addition, the study participants living in urban areas of Kankan and Faranah were more likely to use modern FP methods than those in Conakry (the capital city).

The findings also showed a significant relationship between ethnic group and use of FP methods. Adolescents and young women belonging to the Malinke and Peulh ethnic groups are less likely to use FP methods than those from the Soussou ethnic group. From these findings, we recommend further qualitative research to better understand the reasons why being from these administrative regions and ethnical groups influence use of modern FP methods.

There was no relationship between educational level of urban adolescents and young women and use of FP methods in the multivariate analyses. Contrary to other studies [24, 29, 35] which have found that the likelihood of using a FP methods among female adolescents is significantly associated with the increase in their levels of education.

This study has a number of strengths. First of all, it uses the datasets from the four national representative DHSs conducted in Guinea from 1999 to 2018 and the findings are also based on adequate statistical power (data were weighted for the sampling probabilities) and take account complex sampling procedures during testing of statistical significance. On the other hand, since each panel employed a cross-sectional design, some limitations may affect the conclusions; for example, we cannot determine the temporal relationship between associations with the covariate on use of modern FP methods. Another limitation is also that the factors associated with the use of modern FP methods are based solely on the study of socio-demographic characteristics, which do not include the women’s preferences, experiences with FP use, and behaviors.

## Conclusion

Modern FP use has increased among urban adolescents and youths in Guinea over the past two decades (1999 to 2018). However, there is still a need for efforts to increase enough contraceptive prevalence in these groups. Unmet need for FP among urban adolescents and young women decreased considerably over the time period examined.

Positive predictors of FP use in these groups include being older, wealthier or living in the region of Kankan or Faranah, whereas being married or from the Malinke or Peulh ethnic groups are negative predictors of FP use. This study suggests targeted interventions such as facilitating access adolescent access to FP methods improving awareness raising among the Malinke and Peulh ethnic groups, and improving the regional health systems of Kankan and Faranah to improve access and quality of FP services.

## Data Availability

The datasets used and/or analyzed during the current study are accessible on https://dhsprogram.com/data/available-datasets.cfm

## Abbreviations

AOR: Adjusted odds ratio
CI: Confidence interval
DHS: Demographic and health survey
FP: Family planning
HIV: Human immunodeficiency virus
LMICs: Low- and middle-income countries
STI: Sexually transmitted infection
